# Functional Characterization of a Putative Sortase FA1364 in *Filifactor alocis*

**DOI:** 10.3390/ijms27114783

**Published:** 2026-05-26

**Authors:** Arunima Mishra, Nana Y. Sakyi Opoku, Guangyu Zhang, Richard J. Lamont, Hansel M. Fletcher

**Affiliations:** 1 Division of Microbiology and Molecular Genetics, Department of Basic Sciences, School of Medicine, Loma Linda University, Loma Linda, CA 92350, USA; nsakyiopoku@students.llu.edu (N.Y.S.O.); hfletcher@llu.edu (H.M.F.); 2 Mass Spectrometry Core Facility, Loma Linda University, Loma Linda, CA 92350, USA; guangyuzhang@llu.edu; 3 Department of Oral Immunology and Infectious Diseases, School of Dentistry, University of Louisville, Louisville, KY 40202, USA; rich.lamont@louisville.edu

**Keywords:** *Filifactor alocis*, sortase A, cell wall-anchored proteins, biofilms, coaggregation, periodontal disease

## Abstract

Gram-positive bacteria covalently anchor specific proteins to the peptidoglycan cell wall via sortase, a cysteine transpeptidase that targets proteins with a cell wall sorting signal. Sortase enzymes are critical for bacterial pathogenesis, and their inhibitors have become promising therapeutic targets for infection management. *Filifactor alocis*, a Gram-positive anaerobic bacterium, is now proposed as a diagnostic indicator of periodontal disease. Unlike other bacteria, *F. alocis* encodes a single putative sortase, FA1364. In this study, we functionally characterized the putative sortase FA1364 and found that it belongs to the class A family (SrtA). The SrtA-anchored surface proteins (FA1006, FA1336, FA1424, and FA1750) were identified, and MS/MS analysis confirmed that SrtA is required for their cell-surface localization. The recombinant SrtA protein could recognize and cleave the LPKTG sorting motif with cysteine 191 and arginine 200 as essential catalytic residues. *F. alocis* FLL101 (Δ*FA1364*::*ermF*) showed reduced ability to coaggregate and form biofilm, along with decreased collagen binding and survival in epithelial cells. Additionally, the *FA1364*-defective mutant exhibited increased sensitivity to air exposure. Collectively, these results suggest that the *F. alocis* SrtA protein is an important virulence factor and may represent a novel therapeutic target for the control of periodontal diseases.

## 1. Introduction

*Filifactor alocis* is a newly appreciated, fastidious, gram-positive obligate anaerobe that causes periodontal diseases [1,2]. *F. alocis* was first identified from periodontitis patients as *Fusobacterium alocis* and later, based on 16S rRNA gene sequencing, reclassified into the family Filifactoraceae as *F. alocis* [2]. Periodontal disease is the sixth most common infectious disease worldwide, affecting over 65 million people in the United States, which has also been linked to systemic diseases such as diabetes, coronary heart disease, rheumatoid arthritis, Alzheimer’s disease, and some cancers [3,4,5]. Periodontal disease is associated with a shift in the host inflammatory response, leading to destruction of soft and hard gingival tissues [6,7]. Multiple recent reports have identified *F. alocis* at extra-oral sites in patients with lung cancer, chronic dysphagia, brain abscess, and bacteremia [8,9,10,11]. Additionally, *F. alocis* has been linked to gestational diabetes [12], oral squamous cell carcinoma [13], hypertension in periodontitis patients [14], and the progression of periodontitis in individuals with rheumatoid arthritis [15]. Compared to more traditional periodontal pathogens such as *Porphyromonas gingivalis*, *Treponema denticola,* and *Tannerella forsythia*, *F. alocis* is found at significantly higher levels in diseased individuals and is undetectable in healthy patients [16,17,18]. *F. alocis* possesses virulence attributes consistent with those of a periodontal pathogen, including resistance to oxidative stress [19,20,21,22], ability to modulate host cells [23,24], and survival within neutrophils [25]. Nevertheless, the specific mechanisms and virulence factors that contribute to the pathogenicity of *F. alocis* remain unclear and have yet to be characterized.

**Figure 1:**
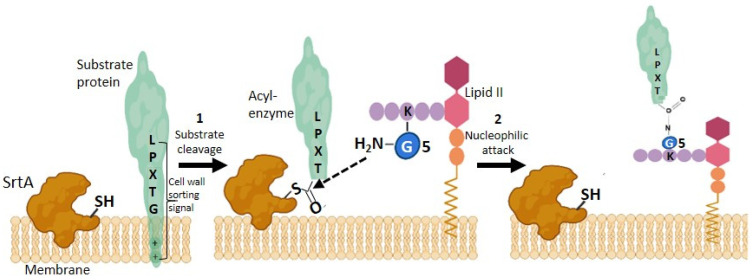
Mechanism of cell wall substrate protein anchoring by class A sortase (SrtA). The SrtA enzyme recognizes the substrate protein containing a cell wall sorting signal and cleaves the LPXTG motif between the threonine (T) and glycine (G) residues to form an acyl-enzyme intermediate. This intermediate is then resolved by nucleophilic attack from the amino group of a peptidoglycan precursor, lipid II, covalently attaching the protein to the peptidoglycan and regenerating SrtA.

Gram-positive bacteria display cell wall-anchored surface proteins, which have been shown to function as important virulence factors essential for host-cell adherence, nutrient uptake, heme transport, sporulation, pilus assembly, and many other housekeeping functions that support bacterial survival in adverse conditions [26]. These proteins are covalently anchored to the cell wall peptidoglycan by membrane-bound cysteine transpeptidase enzymes known as sortase (Srt, MEROPS family C60; EC 3.4.22) [27]. The first sortase enzyme, housekeeping sortase SrtA, was identified in *Staphylococcus aureus*. SrtA substrate proteins have several features at their C-termini (cell wall sorting signal) that are critical for their recognition and cell-surface localization. These include a pentapeptide cell wall sorting motif LPXTG (leucine-proline-any amino acid-threonine-glycine), followed by a hydrophobic region (15–20 amino acids) and a charged tail (a lysine/arginine-rich 5–10 amino acids). In a two-step transpeptidation process, the SrtA enzyme cleaves the LPXTG motif between the threonine and glycine residues, forming an acyl-enzyme intermediate. This intermediate is then resolved by nucleophilic attack from the amino group of a peptidoglycan precursor, lipid II, leading to the covalent attachment of the protein to the peptidoglycan (Figure 1) [27,28].

Based on primary sequences and biological roles, sortase enzymes are grouped into six classes (SrtA to F) [29]. Members of each class recognize a distinct cell wall sorting signal in which the LPXTG motif is varied. Class A sortases are mostly found in low-GC Bacillota (formerly Firmicutes) and perform a housekeeping role in anchoring a variety of functionally distinct surface proteins with an LPXTG recognition motif. Class B enzymes are also predominant in Bacillota, mostly participate in nutrient acquisition, and recognize a (N/S/P)PXTG motif. Class C sortases are found in both Bacillota and Actinobacteria and are responsible for pilus assembly by recognizing the LPXTG motif. Class D sortases display sporulation proteins with a LPXTA recognition motif in *Bacillus* species. Functional roles of Class E and F sortases are not very conclusive [29]. However, studies have shown that class E enzymes may help form aerial hyphae in *Streptomyces coelicolor* and recognize the LAXTG motif [30]. Class F sortases were initially reported in *Cutibacterium acnes* (formerly *Propionibacterium acnes*)*,* which recognizes an LPXTG sorting signal similar to sortase A [31]. Irrespective of their class, all sortases contain a highly conserved catalytic triad His-Cys-Arg, which catalyzes the transpeptidation reaction and the characteristic sortase signature motif, TLXTC, with essential cysteine at the active site for sortase activity [26,32].

*F. alocis* ATCC 35896 possesses one copy of the sortase gene, *HMPREF0389_01364*, which is predicted to encode a putative class C sortase. Currently, there is no information about the *F. alocis* sortase-mediated cell wall-anchored proteins and their role in virulence. This study aims to characterize the putative sortase HMPREF0389_01364 (FA1364), identify its substrates, and determine their role in *F. alocis* virulence. Here, we have determined that contrary to the predicted class C, FA1364 functions as a housekeeping class A sortase (SrtA). We have also identified putative SrtA substrates and, by characterizing the Δ*srtA* mutant, have shown that SrtA-anchored cell wall proteins play an important role in coaggregation, biofilm formation, host cell adhesion, and bacterial survival under aerobic stress. Furthermore, SrtA was overexpressed, purified, and its in vitro cleavage activity confirmed. To our knowledge, this is the first functional characterization of a sortase-deficient mutant from an obligate anaerobe periodontal pathogen.

## 2. Results

### 2.1. In Silico Analysis of F. alocis Putative Sortase *FA1364*

**Figure 2:**
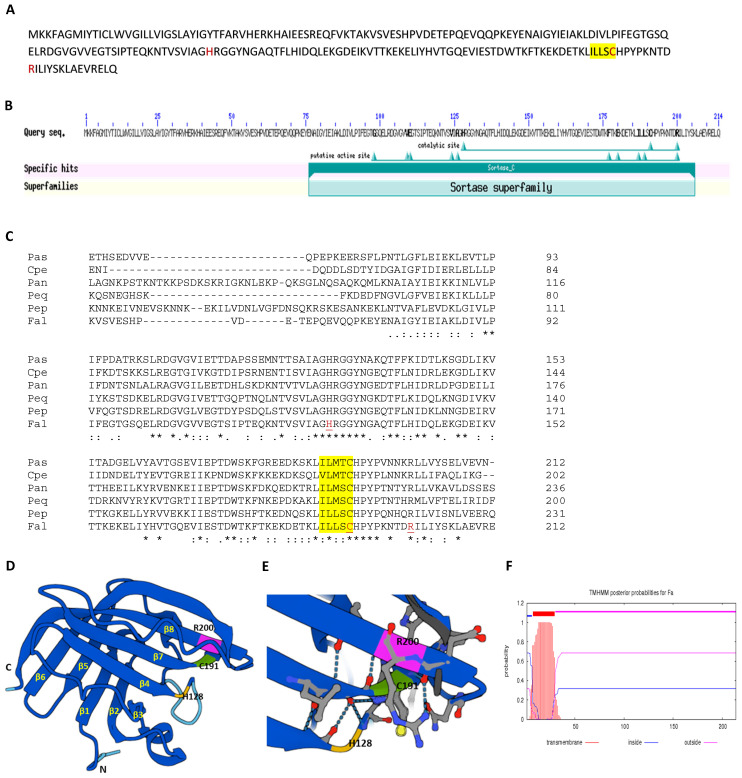
In-silico analysis of *F. alocis* putative sortase FA1364. (**A**) Amino acid sequence of FA1364. Catalytic triad H128C191R200 is shown in red. Sortase signature motif residues ILLSC are yellow highlighted. (**B**) Domain analysis of FA1364 as identified by NCBI blast, version 2.17.0. (**C**) FA1364 multiple sequence alignment with top five sortases using blastP, version 2.17.0. Pas (WP_084231510.1 class C sortase*, Peptoniphilus asaccharolyticus*); Cpe (WP_111694226.1 class C sortase, *Clostridium perfringens*); Pan (WP_002843836.1 class C sortase, *Peptostreptococcus anaerobius*); Peq (WP_269312349.1 class C sortase, *Peptostreptococcus equinus*); Pep (WP_332505701.1 class C sortase, *Peptostreptococcus* sp.); Fal (FA1364 putative class C sortase, *F. alocis*). The conserved sortase signature motifs are yellow highlighted. Sequences were aligned using Clustal Omega 2.0. An asterisk (*) indicates conserved amino acids, a dot (.) indicates amino acids with weakly similar properties and a colon (:) indicates amino acids with strongly similar properties. (**D**,**E**) Alpha-fold 3 model of FA1364_∆N72_ showing catalytic triad H128C191R200. (**F**) Prediction of transmembrane helix using the TMHMM 2.0.

The *FA1364* gene is 645 nucleotides in length and encodes a 214-amino acid protein with a theoretical molecular weight of 24.2 kDa (Figure 2A). According to NCBI *F. alocis* ATCC 35896 genome analysis (https://www.ncbi.nlm.nih.gov/nuccore/NC_016630.1 (accessed on 10 February 2025)), the FA1364 protein belongs to the sortase superfamily and is predicted to encode a class C sortase (Figure 2B). A protein–protein BLAST (Basic Local Alignment Search Tool, version 2.17.0) analysis of FA1364 showed the highest similarity (top 5) with class C sortases from understudied anaerobic bacteria including *Peptostreptococcus equinus*, *P. anaerobius*, *Peptoniphilus asaccharolyticus*, *Peptostreptococcus* species, and *Clostridium perfringens* (48–45% identity). The sequence alignment of FA1364 revealed ILLSC as the sortase signature motif (yellow-highlighted; Figure 2C) instead of the typical TLXTC motif. FA1364 exhibits a conserved catalytic triad (H128C191R200) characteristic of sortases (Figure 2A,C). Bioinformatics analysis of the primary sequence of FA1364 using the TMHMM 2.0 suggested that FA1364 has only one putative transmembrane helix at the N-terminus, which is typical of class A sortases. It lacks the C-terminal hydrophobic domain after the sortase signature motif, a feature characteristic of class C sortases (Figure 2F). The 3D structure of the catalytic domain of FA1364, residues 73–214 (excluding the predicted signal sequence, transmembrane helix, and other disordered regions at the N terminus), was predicted using AlphaFold 3. The predicted structure of FA1364_ΔN72_ revealed the canonical “sortase fold” consist of an elliptical eight-stranded β-barrel architecture. The β-barrel is formed by the interaction of two antiparallel β-sheets (β1, β2, β5, and β6 form one sheet, with β3, β4, β7, and β8 completing the barrel structure). The catalytic residues H128, C191, and R200 are located at the ends of strands β4 and β7 and the beginning of strand β8, respectively (Figure 2D,E). The overall structure is consistent with previously solved sortase A structures [33,34]. Based on these findings, the putative *F. alocis* sortase FA1364 encodes a class A sortase (SrtA) [35,36].

### 2.2. In Silico Identification of the Putative Substrates for F. alocis *SrtA*

**Figure 3:**
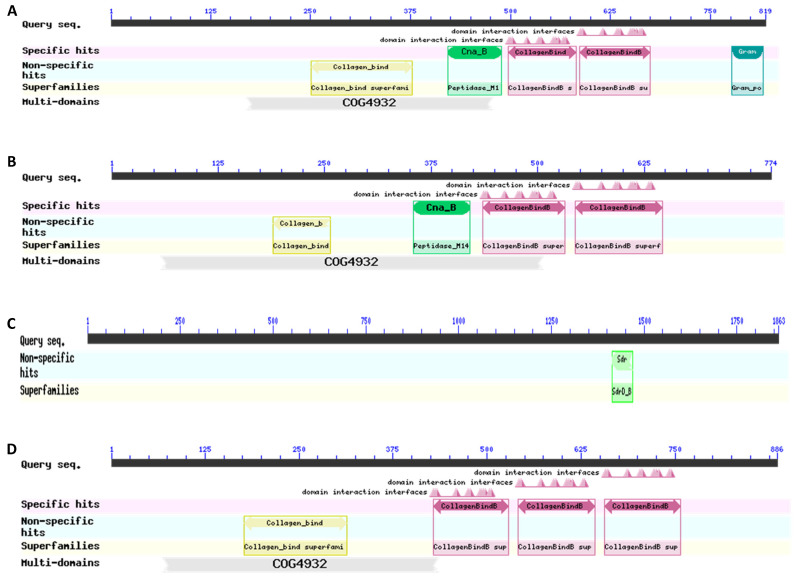
Predicted domain structures of *F. alocis* SrtA substrate proteins. (**A**) FA1006, (**B**) FA1336, (**C**) FA1424, and (**D**) FA1750. Conserved domains were predicted by the NCBI CDD database.

A protein–protein BLAST (version 2.17.0) search for LPXTG/LAXTG/LPXTA motifs in the *F. alocis* ATCC 35896 genome identified a preliminary list of 11 LPXTG containing proteins (accession numbers WP_014263091.1, WP_0156775245.1, WP_014262524.1, WP_014263113.1, WP_041250848.1, WP_014263112.1, EFE28233.1, WP_041250948.1, WP_014262309.1, WP_014261787.1, and WP_014262842.1) as putative SrtA substrates. Of these, six proteins were eliminated because they lacked an N-terminal signal peptide. The rest of the five proteins were examined for the presence of the LPXTG motif within 50 amino acids from the C-terminus. One more protein (WP_014263091.1) was removed from the list because the LPXTG motif was too far (208 amino acids) from the C-terminus. The remaining four proteins, WP_041250848.1 (FA1006), WP_014263112.1 (FA1336), WP_0156775245.1 (FA1424), and WP_014263113.1 (FA1750), containing the LPKTG motif and other characteristics of sortase-anchored cell wall proteins, are shown in Table 1. These proteins are members of the MSCRAMM (microbial surface components recognizing adhesive matrix molecule) family, which has been shown to be a major virulence factor in many Gram-positive bacteria. FA1006, FA1336, and FA1750 contain the Cna_B/collagen binding domain and are putative collagen-binding CNA (collagen adhesin)-like MSCRAMM proteins. FA1424 exhibits a SdrD B-like domain and may be a member of the Sdr (SD-repeat containing) subfamily of the MSCRAMMs (Figure 3).

### 2.3. SrtA Is Required for Cell-Surface Localization of *FA1006*, *FA1336*, *FA1424*, and *FA1750* Proteins

**Figure 4:**
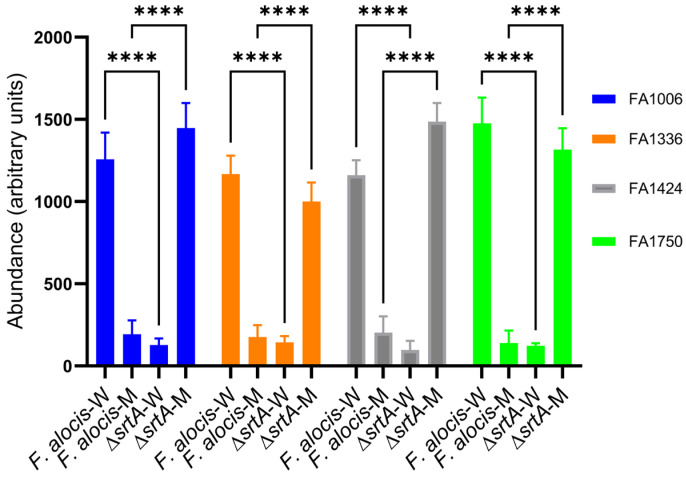
Role of SrtA in cell-surface localization of FA1006, FA1336, FA1424, and FA1750 proteins. *F. alocis* wild-type and ∆*srtA* mutant strains were separated into cell wall (W) and extracellular medium (M) fractions. Protein abundance was determined using LC–MS/MS based label-free quantitation. Data represents three biological repeats. Error bars represent the standard deviations from the means. Statistical analysis was performed using two-way ANOVA followed by Fisher’s LSD post hoc test (**** *p* < 0.0001).

LC–MS/MS-based label-free quantitation (differential analysis) was used to measure the abundance of sortase substrate proteins FA1006, FA1336, FA1424, and FA1750 in the cell wall and extracellular medium fractions of wild-type and ∆*srtA* mutant strains. An isogenic mutant of *F. alocis* ATCC 35896 was created by replacing the *FA1364* gene with an erythromycin resistance cassette (*ermF*). After the electroporation of *F. alocis* with the purified fused PCR fragment, two colonies were detected on erythromycin plates after five days of incubation. The replacement of the *FA1364* gene with the *ermF* cassette in these mutants was confirmed by PCR and DNA sequencing (Supplementary Figure S1 in the Supplementary Materials). The isogenic mutant, designated FLL101 (Δ*FA1364*::*ermF*) was selected for further studies. In the wild-type strain, the FA1006, FA1336, FA1424, and FA1750 proteins were significantly more abundant in the cell wall fraction compared to the extracellular medium (Figure 4). The subcellular localization of the proteins was significantly affected by the absence of sortase. In the ∆*srtA* mutant, the substrate proteins were significantly more abundant in the culture medium compared to the cell wall fraction (Figure 4). These results confirm the role of SrtA in attaching the FA1006, FA1336, FA1424, and FA1750 proteins to the cell surface.

### 2.4. *SrtA* Contributes to the Binding of F. alocis to Collagen

**Figure 5:**
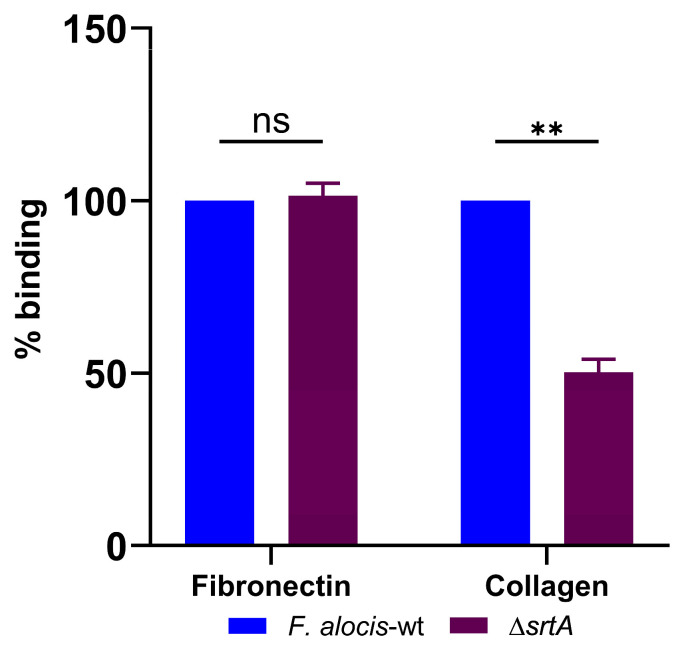
Binding of *F. alocis* wild-type and isogenic ∆*srtA* mutant to collagen and fibronectin. *F. alocis* cultures, grown overnight in BHI broth and suspended in PBS to an OD_600_ of 1.0, were allowed to bind on collagen and fibronectin coated 96-well plates for 4 h. After incubation, the bound bacteria were detached, appropriately diluted, plated on BHI agar plates, and incubated anaerobically for 5-7 days at 37 °C. The percentage of bacterial binding was determined by comparing the CFU/mL of adherent bacteria for each strain with input numbers. Experiments were carried out in three independent repeats in triplicates. Error bars represent the standard deviations from the means. Statistical analysis was performed using unpaired *t*-test (** *p* < 0.005; ns, nonsignificant).

Considering that three SrtA substrates are putative collagen adhesion proteins, we examined the effect of the ∆*srtA* mutant on cellular collagen binding. We evaluated the binding of wild-type *F. alocis* and the ∆*srtA* isogenic mutant to collagen type I and fibronectin, used as a control. The *srtA*-deficient mutant showed significantly reduced binding to collagen as compared to the wild-type strain (~50% versus 100% binding for wild-type, Figure 5). In contrast, as a control, both wild-type and ∆*srtA* mutant strains showed no difference in fibronectin binding (Figure 5). These results suggest that SrtA-anchored collagen-binding adhesins FA1006, FA1336, and FA1750 may contribute to the binding of *F. alocis* to collagen.

### 2.5. Coaggregation Ability of F. alocis Wild-Type and *Δ*srtA Mutant

**Figure 6:**
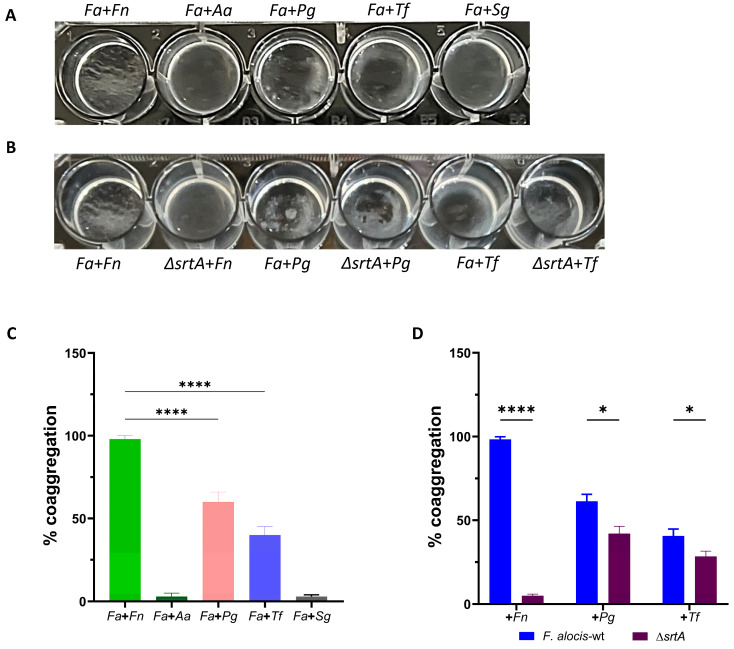
Coaggregation ability of *F. alocis* wild-type and Δ*srtA* mutant. Stationary-phase cultures of bacterial strains, *F. alocis* wild-type (*Fa*), *F. alocis* Δ*srtA* mutant (∆*srtA*), *F. nucleatum* (*Fn*), *A. actinomycetemcomitans* (*Aa*), *P. gingivalis* (*Pg*), *T. forsythia* (*Tf*), and *S. gordonii* (*Sg*) were harvested, washed in coaggregation buffer and normalized to an OD_600_ of 1.0. Then, 1 mL aliquots of bacterial cell suspensions were mixed together. (**A**,**B**) Coaggregated bacteria were photographed in a 24-well plate 30 min after the assay. (**C**,**D**) Coaggregation percentage was determined by measuring OD_600_ after 30 min of incubation without agitation at room temperature. The results represent the means of three independent experiments. Error bars represent the standard deviations from the means. Statistical analysis was performed using one-way and two-way ANOVA for C and D, respectively, followed by Fisher’s LSD post hoc test (**** *p* < 0.0001, * *p* < 0.05).

Initially, we investigated the coaggregation ability of *F. alocis* wild-type with known oral bacteria, including *Fusobacterium nucleatum*, *Aggregatibacter actinomycetemcomitans*, *P. gingivalis*, *T. forsythia*, and *Streptococcus gordonii*. As shown in Figure 6A,C, wild-type *F. alocis* strongly coaggregated with *F. nucleatum* (~100%), followed by reduced coaggregation with *P. gingivalis* (~61%) and *T. forsythia* (~40%). It did not coaggregate with *A. actinomycetemcomitans* and *S. gordonii* (Figure 6A,C). Furthermore, the coaggregation ability of the Δ*srtA* mutant was examined with *F. nucleatum*, *P. gingivalis*, and *T. forsythia*. Compared to the wild-type strain, the *F. alocis* Δ*srtA* mutant displayed little to no coaggregation with *F. nucleatum* (~5 versus ~100%) and had significantly reduced coaggregation with *P. gingivalis* (42 versus 61%), and *T. forsythia* (28 versus 40%) (Figure 6B,D). These results suggest that deletion of *srtA* impacts the coaggregation ability of *F. alocis* with *F. nucleatum*, *P. gingivalis*, and *T. forsythia*.

### 2.6. Biofilm Formation by F. alocis Wild-Type and the *Δ*srtA Isogenic Mutant

**Figure 7:**
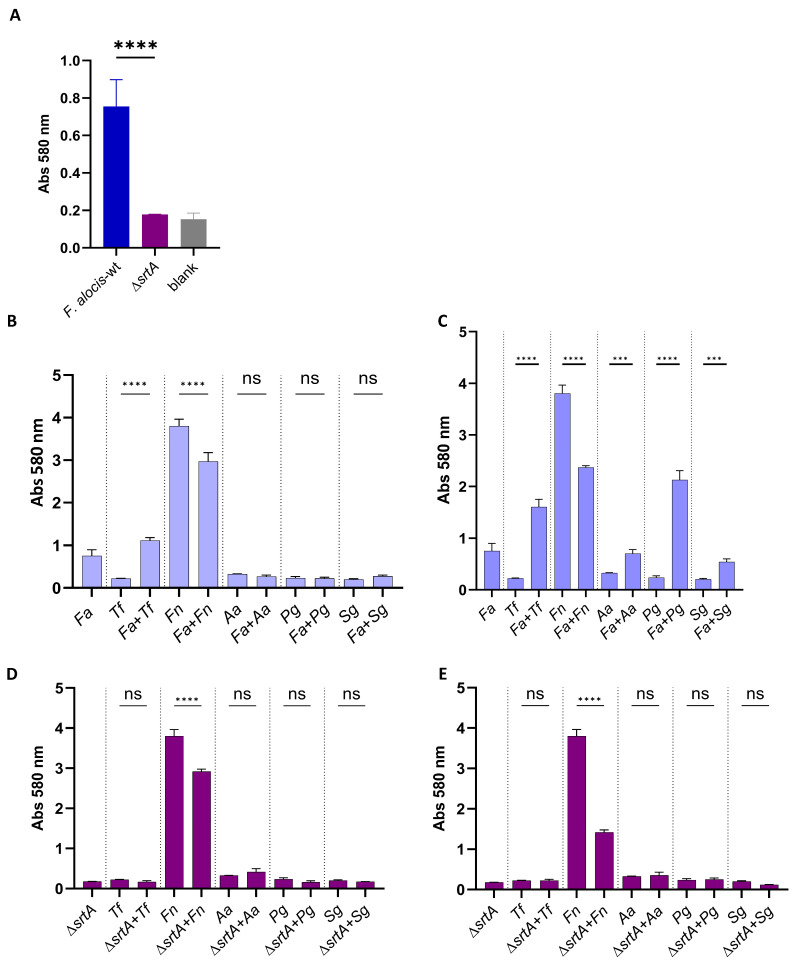
Role of *F. alocis* wild-type and Δ*srtA* mutant in in vitro biofilm formation. (**A**) 200 μL of *F. alocis* wild-type and Δ*srtA* mutant cultures, (**B**,**D**) Either 200 μL of individual cultures of *F. alocis* (*Fa*), *srtA* deficient mutant of *F. alocis* (Δ*srtA*), *T. forsythia* (*Tf*), *F. nucleatum* (*Fn*), *A. actinomycetemcomitans* (*Aa*), *P. gingivalis* (*Pg*) and *S. gordonii* (*Sg*) or 100 μL of cocultured bacteria were transferred to 96-well plate. (**C**,**E**) 100 μL of *Tf, Fn*, *Aa, Pg* and *Sg* cultures (OD_600_ ~0.05) were transferred on top of 100 μL of *F. alocis* wild-type and/or Δ*srtA* mutant cultures (OD_600_ ~0.05) grown in 96-well plates for 24 h. All plates were incubated at 37 °C inside the anaerobic chamber. Bacterial biofilms were stained with 0.5% crystal violet and quantitated by measuring the absorbance at 580 nm. BHI broth was used as blank. The values presented here are the means of four independent experiments. Error bars represent the standard deviations from the means. Statistical analysis was performed using one-way ANOVA followed by Fisher’s LSD post hoc test (**** *p* < 0.0001; *** *p* < 0.0005; ns, nonsignificant).

Since biofilm formation is an adhesin-dependent process, an in vitro biofilm assay was used to assess the ability of *F. alocis* wild-type and Δ*srtA* mutant strains to form mono-species and dual-species biofilm with *T. forsythia*, *F. nucleatum*, *A. actinomycetemcomitans*, *P. gingivalis*, and *S. gordonii*. Under tested conditions, *F. nucleatum* displayed the greatest biofilm-forming ability in a mono-species biofilm, followed by *F. alocis*, *A. actinomycetemcomitans*, *P. gingivalis*, *T. forsythia,* and *S. gordonii*. In dual-species biofilms grown in coculture, wild-type *F. alocis* significantly enhanced *T. forsythia* biofilm formation but had no effect on *P. gingivalis*, *S. gordonii*, or *A. actinomycetemcomitans*. *F. nucleatum* produced less biofilm (~78% versus 100%) in coculture with *F. alocis* (Figure 7B). When these bacteria were grown on top of overnight-cultured *F. alocis*, their biofilm-forming ability was significantly increased, except for *F. nucleatum* (Figure 7C). To examine the role of Δ*srtA* in mono-species biofilm formation, both wild-type *F. alocis* and the Δ*srtA* mutant were cultured in 96-well plates to evaluate their biofilm-forming capacity. As shown in Figure 7A, biofilm formation by the Δ*srtA* mutant was significantly reduced compared to the wild-type strain. This decrease was not due to reduced growth as the Δ*srtA* mutant had the same cell density (OD_600_) as the wild-type *F. alocis* when cultured in 96-well plates (OD_600_ = 0.27 for wild-type *F. alocis* and 0.262 for the Δ*srtA* mutant). The Δ*srtA* mutant did not affect the biofilm-forming ability of *P. gingivalis*, *S. gordonii*, *T. forsythia* or *A. actinomycetemcomitans*. Compared to the wild-type *F. alocis*, the mutant significantly decreased the biofilm formation in *F. nucleatum* under both conditions (Figure 7D,E). These results demonstrate that deletion of *srtA* impacts the in vitro biofilm formation of *F. alocis* and may affect the biofilm-forming ability of *F. nucleatum*.

### 2.7. Role of *SrtA* in Survival of F. alocis in Host Cells

**Figure 8:**
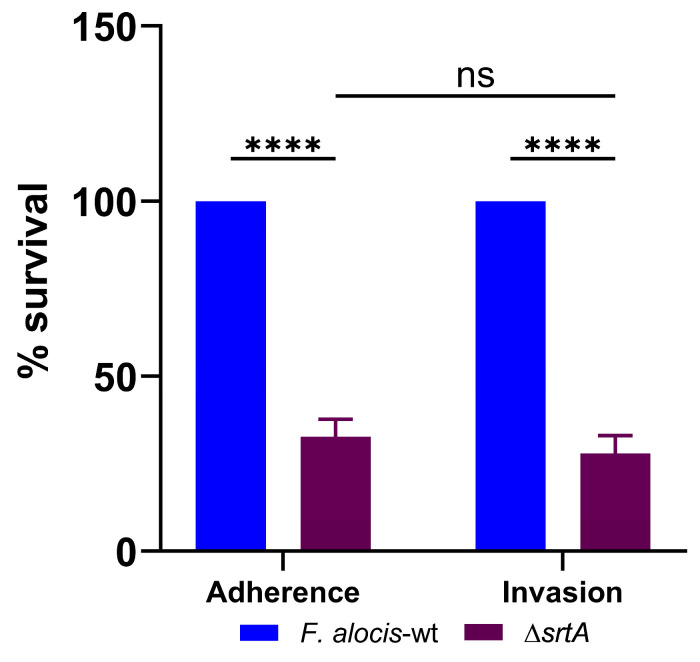
Adherence and invasion of *F. alocis* wild-type and Δ*srtA* mutant in TIGK cells. Cells were grown and maintained at 37 °C under 5% CO_2_ in supplemented DermaLife K Serum-Free Keratinocyte Culture Medium. The 10^5^ Epithelial cells were infected with 10^7^ bacteria (MOI:100) for 1 h in an anaerobic chamber. For the invasion assay, extracellular bacteria were killed by incubating the cells with 200 μg/mL of metronidazole for 1 h. The percent adhesion and invasion were calculated, and data are presented as percentage to that of wild-type, which was set to 100%. Experiments were done in triplicates in three independent repeats. Error bars represent the standard deviations from the means. Statistical analysis was performed using two-way ANOVA followed by Fisher’s LSD post hoc test (**** *p* < 0.0001; ns, nonsignificant).

Cell-surface MSCRAMMs of gram-positive bacteria play an important role in adhesion to host tissues, a crucial step in disease initiation. Gingival epithelial cells are among the first host cells encountered by periodontal bacteria. To explore how the SrtA-anchored cell surface proteins affect bacterial virulence, the adhesion and invasion abilities of *F. alocis* wild-type and ∆*srtA* mutant in TIGK cells were assessed. As shown in Figure 8, the mutant exhibited a significant reduction in its ability to adhere and invade to TIGK cells compared with the wild-type strain. After 1 h of incubation, the adhesion and invasion rate of the ∆*srtA* mutant was only 32% and 28% of the wild-type *F. alocis,* which was set to 100%. The invasion ability of the mutant was not significantly different than its adhesion (28% invasion compared to 32% adhesion) (Figure 8). These results suggest that SrtA-anchored cell-surface proteins may contribute to *F. alocis* adhesion to gingival epithelial cells.

### 2.8. Sensitivity of *Δ*srtA Mutant to Atmospheric Air Exposure

**Figure 9:**
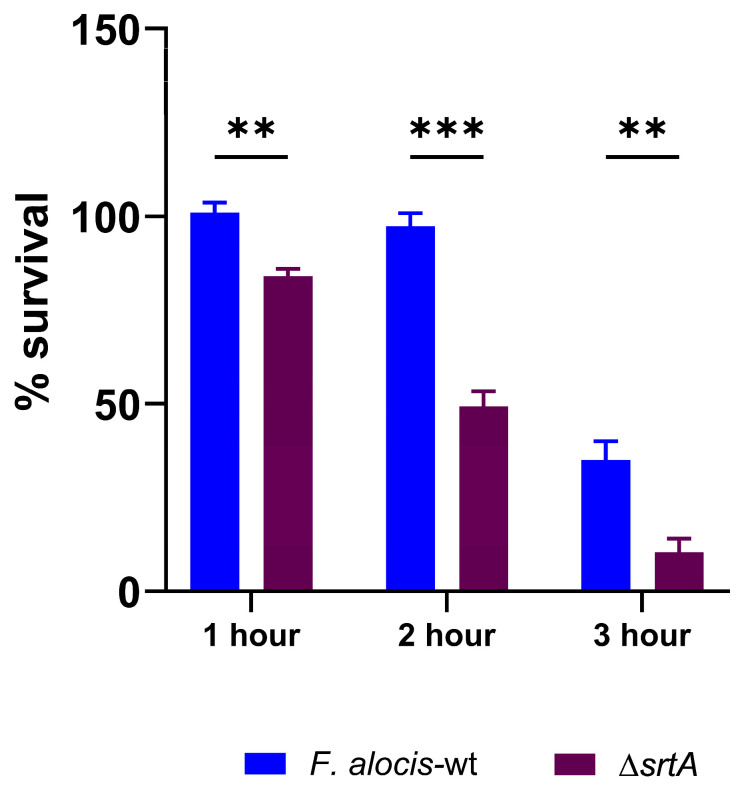
Survival of *F. alocis* wild-type and Δ*srtA* mutant strains exposed to atmospheric air. Bacteria on BHI agar plates were exposed to air for the indicated time periods, the plates were then transferred back to the anaerobic chamber, incubated at 37 °C, and colonies were counted after 5–7 days. CFU/mL were determined and percent survival was calculated. The results represent the means of three independent experiments. Error bars represent the standard deviations from the means. Statistical analysis was performed using unpaired *t*-test (*** *p* < 0.0001, ** *p* < 0.005).

The relative resistance of *F. alocis* to oxidative stress is a key adaptive trait that allows it to survive in the reactive oxygen species-rich microenvironment of the periodontal pocket [22]. The expression of bacterial adhesins and oxidative stress response genes can be co-regulated by global regulators, enabling pathogens to defend against oxidative stress while also enhancing adherence for colonization [37,38]. To assess the role of SrtA in oxidative stress resistance, both wild-type *F. alocis* and Δ*srtA* mutant strains were exposed to air, and their percent survival was evaluated. As shown in Figure 9, the mutant was significantly more sensitive to air exposure compared to the wild-type *F. alocis* strain. When grown on BHI agar plates, the wild-type *F. alocis* strain showed nearly 100% survival after 1 and 2 h of air exposure, whereas after 3 h the survival rate was 35%. In contrast, following 1, 2, and 3 h of air exposure, the survival rates were 84%, 49%, and 10%, respectively, for the Δ*srtA* mutant strain (Figure 9). These findings imply that deletion of *srtA* may impair *F. alocis* survival in the presence of atmospheric air.

### 2.9. Enzymatic Activity of F. alocis *SrtA*

**Figure 10:**
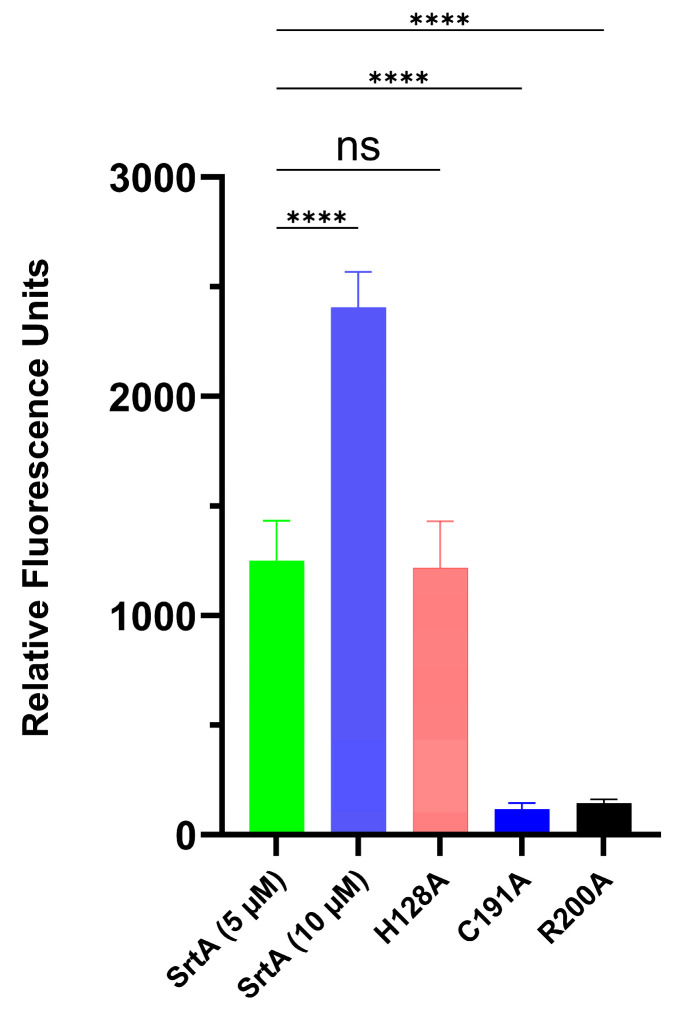
Enzymatic activity of *F. alocis* SrtA. The in vitro activity of the recombinant SrtA_△N72_ and the mutant enzymes (H128A, C191A and R200A) was performed in 96-well black, clear-bottom plate in a final volume of 200 μL containing 150 mM NaCl, 5 mM CaCl_2_, 50 mM Tris–HCl, pH 7.5, 5/10 µM wild-type or 5 µM mutant proteins, and 20 μM of Dabcyl-RHLPKTGDG-Edans peptide at 37 °C. Peptide cleavage was measured after 1 h as an increase in relative fluorescence units with excitation and emission wavelengths set to 350 nm and 495 nm, respectively. The assay was done in triplicates in three independent experiments. Statistical analysis was performed using one-way ANOVA followed by Fisher’s LSD post hoc test (**** *p* < 0.0001; ns, nonsignificant).

To assess the cleavage activity of the purified recombinant *F. alocis* SrtA_ΔN72_ protein on the predicted LPKTG motif, a fluorescent-labeled peptide containing the LPKTG sorting motif was used as the substrate (Dabcyl-RHLPKTGDG-Edans). The fluorescence of the Edans fluorophore within the peptide is quenched when in close proximity to Dabcyl. When the substrate peptide is cleaved by sortase between the threonine and glycine residues, the Edans fluorophore is released from Dabcyl, resulting in a detectable fluorescent signal. As shown in Figure 10, an increase in fluorescence (RFUs) was observed over time when the wild-type SrtA_ΔN72_ protein was incubated with the peptide, indicating that the LPKTG peptide cleavage occurred in the presence of SrtA_ΔN72_ over 1 h. Fluorescent intensity increased proportionally when the enzyme concentration was doubled (Figure 10).

To characterize the role of the conserved His-Cys-Arg catalytic triad in SrtA activity, mutants replacing H128, C191, and R200 with alanine were created through site-directed mutagenesis, and their catalytic activities were assessed as described above. The active site cysteine and arginine mutant proteins (SrtA_ΔN72, C191A_ and SrtA_ΔN72, R200A_) showed significantly reduced cleavage activity compared to the wild-type SrtA_ΔN72_ enzyme. In contrast, the cleavage activity of the histidine mutant protein (SrtA_ΔN72, H128A_) was similar to that of the wild-type (Figure 10). Together, these results indicate that SrtA_ΔN72_ can recognize and cleave the LPKTG motif and the C191 and R200 residues are essential for its activity.

## 3. Discussion

All Gram-positive bacteria possess transpeptidase enzymes, commonly known as ‘‘sortases” [28,29]. These enzymes play a key role in regulating bacterial cell surface structures by covalently attaching various proteins to the cell walls [26]. Sortase-anchored proteins in pathogenic bacteria are essential for adhesion and pathogenicity, immune modulation, biofilm development, heme uptake, and the acquisition of essential nutrients [29]. Therefore, sortases are a vital virulence factor and could be targeted with drugs to combat bacterial infections [39]. Bacteria often encode multiple sortases, with the number varying among species. Unlike many other Gram-positive bacteria, *F. alocis* ATCC 35896 carries a single putative sortase, FA1364, predicted to be a class C sortase. Class C sortases are pilus/fimbria-specific and are primarily responsible for polymerizing bacterial pilin or fimbrial subunits [29]. Genes for pilin subunits and class C sortase are usually found together in the genome as a single operon to ensure coordinated expression [29,35]. In contrast, genes encoding class A sortases are generally not clustered with their substrates, which include a variety of surface proteins with diverse biological functions. No pilus or fimbrial gene homolog has been identified in the *F. alocis* genome (https://www.ncbi.nlm.nih.gov/nuccore/NC_016630.1 (accessed on 10 February 2025)). Additionally, the *FA1364* gene is not located within an operon with any of its substrate-encoding genes. Sortase classification relies solely on phylogenetic analysis and primary sequence [35,36]. The main difference between class C and A sortases is the presence of a C-terminal hydrophobic domain following the sortase signature motif TLXTC in class C [36]. Sequence analysis of FA1364 did not show a C-terminal hydrophobic domain after the ILLSC signature motif. This data indicates that FA1364 is likely a housekeeping class A sortase [35,36]. Since the primary function of a housekeeping sortase is to anchor its substrates to the cell wall, the phenotype of the ∆*srtA* mutant could be examined with the knowledge of the functions of the substrate proteins. Therefore, our first goal was to identify the SrtA-anchored surface proteins and then to determine their localization in the sortase-deficient ∆*srtA* mutant. A protein–protein BLAST (version 2.17.0) search for SrtA sorting signal LPXTG in the *F. alocis* genome identified four substrate proteins, FA1006, FA1336, FA1424, and FA1750, all containing the LPKTG motif and predicted to belong to the MSCRAMM family. Blast (version 2.17.0) parameters were adjusted for short input sequences to ensure not to miss any target protein utilizing different matrices and a range of expect threshold values. MSCRAMMs are ligand-binding proteins that play a critical role in the virulence modulation of Gram-positive bacteria by mediating pathogen attachment to and colonization of host tissues which is an early step in infection [40]. FA1006, FA1336, and FA1750 are annotated as CnaB-type domain-containing proteins and are putative collagen adhesins (CNA). CNA is a collagen-binding protein first identified in *S. aureus* and was shown to bind soluble collagen type I [41]. Since then, CNA homologs have been identified in several Gram-positive bacteria, including *Enterococcus faecalis* [42], *Enterococcus faecium* [43], *Streptococcus mutans* [44], and *Bacillus anthracis* [45]. Most CNA proteins have been shown to act as virulence factors by targeting collagen thereby enhancing bacterial adhesion to host tissues during infection [40]. FA1424 is annotated as a SdrD B-like domain protein. Sdr proteins constitute a subfamily of MSCRAMM that includes members such as clumping factor A (ClfA), ClfB, SdrC, SdrD, and SdrE in *S. aureus*, as well as SdrF and SdrG in *Staphylococcus epidermidis* [46]. All Sdr proteins consist of a N-terminal signal peptide, followed by a ligand-binding A domain, 1–5 spacer-like B repeats, and the characteristic R region containing serine-aspartate (SD) repeats. Unlike SdrD, which has 132–170 SD repeats, FA1424 did not appear to have any SD repeats in its primary sequence and shares homology only with the structural domain of SdrD. Therefore, identifying FA1424 as a Sdr-like protein may be premature, and it could be considered a hypothetical protein in *F. alocis*.

Consistent with the loss of function of SrtA-anchored surface MSCRAMMs, the ∆*srtA* mutant showed significantly reduced binding to collagen, decreased coaggregation, impaired biofilm formation and lower adhesion to gingival cells in vitro. In particular, the Δ*srtA* mutant significantly decreased the biofilm formation in *F. nucleatum* but did not affect the biofilm-forming ability of *P. gingivalis*, *S. gordonii*, *T. forsythia* or *A. actinomycetemcomitans*. Bacterial inter-species coaggregation plays an important role in biofilm formation. Coaggregation promotes the spatial organization of bacteria within biofilms, increases bacterial survival and modulates virulence factor expression [47]. *F. alocis* is shown to tightly colocalize and coaggregate with *F. nucleatum* during the establishment and maturation of the oral biofilms [48]. Our results also confirm the strong coaggregation between *F. alocis* and *F. nucleatum*. The inability of the Δ*srtA* mutant to coaggregate with *F. nucleatum* may likely affect the biofilm-forming ability of this bacterium. It may also suggest that one of the SrtA attached cell surface adhesins in *F. alocis* is required for coaggregation with *F. nucleatum* and could involve *F. nucleatum* adhesins Fap2 or FadA [47]. Future studies requiring individual *F. alocis* cell surface adhesin mutants ∆*FA1006*, ∆*FA1336*, ∆*FA1424*, and ∆*FA1750* are necessary to confirm this.

To further verify the results of the Δ*srtA* mutant, it would be desirable to complement the defective *srtA* gene in the mutant to restore the wild-type phenotype. The genetic system in *F. alocis* is underdeveloped and therefore, complementation of the ∆*srtA* mutant was not feasible at this time. Currently, there is no *E. coli*/*F. alocis* shuttle vector available to do complementation in *Filifactor*. Preliminary assessment of several *F. alocis* strains in our laboratory has not identified any plasmids. Because *Filifactor* is distantly related to *Clostridium*, plasmids belonging to the genus *Clostridium*, such as pJIR750 (chloramphenicol resistant) and pJIR751 (erythromycin resistant) were evaluated for complementation purposes in *Filifactor*. Attempts to use pJIR750 plasmid itself and to replace *ermF* cassette in the mutant with wild-type copy of *srtA* gene fused to chloramphenicol resistant cassette were unsuccessful. Given the limited number of available antibiotic resistance markers for *F. alocis*, a markerless gene-deletion system would be advantageous. For a markerless mutant, erythromycin, which is the only functional marker for *Filifactor*, could be used for complementation studies. Work to improve *F. alocis* genetic system is ongoing in the laboratory.

One intriguing feature of the ∆*srtA* mutant is its sensitivity towards air exposure. None of the SrtA substrates we identified is predicted to play a role in the oxygen detoxification pathways, suggesting that this may be a common feature of housekeeping sortases in periodontal bacteria, or specific to *F. alocis* SrtA. To date, none of the *srtA* mutants from obligate anaerobe periodontal bacteria have been characterized. As mentioned above, FA1424 appears to be a hypothetical protein and might be responsible for *F. alocis* survival in the presence of air, similar to superoxide reductase [19]. A comprehensive analysis, involving mutants deficient in each of the SrtA substrate proteins, is necessary to confirm this hypothesis and is the subject of further investigation in the laboratory. Alternatively, we cannot rule out that the expression of bacterial adhesins and the response to oxidative stress may involve pathways co-regulated by global regulators [37,38]. In *A. actinomycetemcomitans*, ArcA, the response regulator of the ArcAB two-component system involved in redox homeostasis signaling, has been implicated in controlling the expression and function of extracellular matrix protein adhesin A (EmaA), a major surface adhesin essential for biofilm formation and the initiation of pathogenic processes [38]. In addition, the oxidation-sensing regulator AbfR in *S. epidermidis* is upregulated under H_2_O_2_-induced oxidative stress and directly influences both the oxidative stress response and bacterial aggregation/biofilm formation [37]. Also, members of the MarR family, such as MgrA, utilize cysteine oxidation in *S. aureus* to regulate virulence, including adhesin expression [49]. It is unclear if a similar mechanism is functional in *F. alocis* and is the subject of further investigation.

Once the putative SrtA substrate proteins were identified, we assessed the impact of sortase deficiency on the anchoring of proteins FA1006, FA1336, FA1424, and FA1750 to the cell surface. Previously, gel-based approaches were commonly used to evaluate the impact of sortase gene deletion on the subcellular localization of substrate proteins. However, these experiments showed practically identical protein bands in the cell wall extracts from both the wild-type and ∆*srtA* mutant strains [50,51], suggesting that gel-based approaches cannot distinguish covalently attached surface proteins between the wild-type and mutant strains. In fact, cell wall and extracellular culture medium fraction proteins from *F. alocis* wild-type and ∆*srtA* mutant strains were indistinguishable on SDS-PAGE (Supplementary Figure S2 in the Supplementary Materials). Therefore, we used LC–MS/MS-based label-free quantitation to measure the levels of covalently attached FA1006, FA1336, FA1424, and FA1750 proteins in the cell wall and extracellular medium fractions of wild-type and mutant strains. These experiments showed high levels of these proteins in the cell wall of the *F. alocis* wild-type and in the extracellular medium of the mutant, confirming SrtA’s role in surface localization of these proteins. We also observed small amounts of these proteins in the mutant cell wall. Our results are consistent with earlier results obtained for *S. gordonii,* where the LPXTG proteins, SspA and SspB from the *srtA* mutant, were mostly found in the extracellular medium, but still expressed on the cell surface in reduced amounts [50]. Previously, a *S. aureus srtA* mutant was shown to accumulate nonfunctional surface protein precursors in the cell wall [52], which may explain the low protein levels observed in the cell wall fraction of the ∆*srtA* mutant in *F. alocis*. It is noteworthy that we also observed a high abundance of a few other proteins in the extracellular fraction of the ∆*srtA* mutant, which lacked the C-terminal sorting signal LPXTG. We cannot rule out the possibility that the location of these proteins may be indirectly affected by sortase activity. This is a subject of ongoing investigation in the lab.

Despite several attempts, we were unable to express the full-length *F. alocis* SrtA. SrtA proteins are membrane-bound with an N-terminal signal peptide and an N-terminal hydrophobic transmembrane anchor, which makes them unstable and difficult to purify. To address these challenges, truncated SrtA_ΔN72_ was expressed by removing the first 72 N-terminal amino acid residues, which include the predicted signal sequence and other N-terminus disordered regions. The truncated enzymes have been shown to retain their native transpeptidase activity [34,53,54]. The catalytic activity of SrtA_ΔN72_ was confirmed by using a fluorescent-labeled peptide with the LPKTG motif. The active site C191A and R200A mutants exhibited no activity, which is consistent with the observations reported earlier, indicating the indispensability of active site cysteine and arginine residues for any sortase activity [53,55,56]. The H128A mutant showed a comparable level of activity to the wild-type enzyme. The corresponding active site histidine residue in *S. aureus* SrtA, H130, has been demonstrated to be essential for its activity [53], whereas, similar to our study, the corresponding H116 of *Clostridium difficile* SrtB did not affect its catalytic activity [56]. Overall, for sortase enzymes, the conserved histidine acts as a general base to activate the cysteine thiol [57]. Our results may suggest the presence of an alternative proton acceptor or a water-assisted activation pathway that could compensate for the loss of H128. Alternatively, *F. alocis* SrtA may use a yet unidentified basic residue in place of histidine to stabilize the thio-acyl intermediate. Confirmation of these hypotheses requires further investigation involving the detailed structure analysis of SrtA in *F. alocis*.

Since the pioneering work on SrtA and its role in the pathogenesis of *S. aureus* infections, SrtA inhibitors have been an active area of research, with several studies exploring their use to reduce bacterial virulence by preventing host tissue adherence and disrupting biofilm formation [54,58]. SrtA is readily accessible in the cell membrane and is not essential for bacterial growth, making it an ideal target for anti-virulence drug development. Compared with conventional antibiotics and other anti-virulence strategies, the main advantage of SrtA inhibitors is that they target bacterial virulence rather than killing bacteria. Future studies evaluating various types of SrtA inhibitors, including synthetic small molecules, peptides, and other natural products, against *F. alocis* SrtA, a likely drug target for the control of periodontal diseases, are ongoing in the laboratory.

## 4. Materials and Methods

### 4.1. Bacterial Strains, Plasmids and Growth Conditions

All bacterial strains and plasmids used in this study are listed in Table 2. The *F. nucleatum*, *P. gingivalis*, *S. gordonii*, and *A. actinomycetemcomitans* strains were cultured in Brain Heart Infusion (BHI) broth supplemented with yeast extract (0.5%), hemin (5 µg/mL), vitamin K (0.5 µg/mL), and cysteine (0.1%). *F. alocis* strains were grown in BHI broth supplemented with yeast extract, hemin, vitamin K, cysteine, and L-arginine (100 µM). *T. forsythia* was grown in BHI broth supplemented with yeast extract (0.5%), hemin (5 µg/mL), vitamin K (0.5 µg/mL), cysteine (0.1%), N-acetyl muramic acid (0.001%), and fetal bovine serum (5%). *Escherichia coli* strains were grown aerobically in Luria-Bertani (LB) broth with shaking. *F. alocis*, *F. nucleatum*, *P. gingivalis*, *S. gordonii*, and *T. forsythia* were cultured in an anaerobic chamber (Coy Manufacturing, Grass Lake, MI, USA) in 10% H_2_, 10% CO_2_, and 80% N_2_. *A. actinomycetemcomitans* was cultured under microaerophilic conditions. Bacterial growth rates were determined spectrophotometrically by measuring optical density at 600 nm (OD_600_). Antibiotics erythromycin (5 µg/mL) and ampicillin (100 µg/mL) were used as needed. All organisms were incubated at 37 °C. BHI broth, yeast extract, LB broth, and fetal bovine serum were purchased from Thermo Fisher Scientific, Waltham, MA, USA. Hemin, vitamin K, cysteine, L-arginine, N-acetyl muramic acid, erythromycin, and ampicillin were purchased from Sigma–Aldrich, St. Louis, MO, USA.

### 4.2. Identification of F. alocis Putative Cell Wall-Anchored Proteins

The genome of *F. alocis* ATCC 35896 was searched for proteins containing LPXTG, LAXTG, or LPXTA motifs using the BLAST (version 2.17.0) search tool (matrix PAM30, expect threshold value 25 and word size 2) [62] from the National Center for Biotechnology Information (NCBI). The resulting proteins were further refined by excluding those without an N-terminal signal sequence as predicted by SignalP 6.0, or if the motif was not located within the 60–70 amino acids of the C-terminus. Identified proteins were assigned putative functions based on their conserved domains, which were determined using the NCBI Conserved Domain Search.

### 4.3. Construction of F. alocis *FLL101* (*Δ*FA1364::ermF) Mutant

The construction of the *F. alocis* isogenic *srtA* mutant was performed using a long PCR-based fusion method, as previously described with minor modifications [19]. Briefly, approximately 600 bp upstream and downstream fragments of the *srtA* gene were PCR amplified from *F. alocis* parental chromosomal DNA. The promoterless *ermF* cassette without a transcriptional terminator was amplified from the plasmid pVA2198 with primers containing overlapping bases for the upstream and downstream fragments of *srtA,* respectively. Next, the upstream fragment, *ermF*, and downstream fragments were fused together, and the purified fusion product (~2.0 kb) was electroporated into *F. alocis* competent cells, which were then plated on BHI agar plates containing 5 µg/mL of erythromycin and incubated at 37 °C for 5–7 days inside the anaerobic chamber. Erythromycin-resistant colonies were screened for replacement of *srtA* with *ermF* by PCR and confirmed by DNA sequencing. The primers used in this study are listed in Table 3. Go Taq Green Master Mix for PCR fusion reaction was purchased from Promega, Madison, WI, USA.

### 4.4. Electroporation of F. alocis

Electroporation of *F. alocis* was performed as previously described with minor modifications [19]. Briefly, a 2 mL overnight culture was used to inoculate 8 mL of fresh BHI broth, which was then incubated at 37 °C in the anaerobic chamber. After 24 h, the cells (OD_600_ ~0.24) were harvested by centrifugation at 4 °C, washed twice, resuspended in 1 mL of ice-cold electroporation buffer (10% glycerol, 1 mM MgCl_2_), and incubated on ice for 15 min. The cells were centrifuged again and washed with 1 mL of 10% glycerol and resuspended in 200 µL of 10% glycerol; 1–2 µg of DNA (fusion product) was added to the 100 µL competent cells. The mixture was placed in a sterile 0.2 cm gap cuvette, incubated on ice for 10 min and pulsed with a gene pulser at 2.5 volts and 600 ohms (Gene Pulser Xcell #1652660, Bio-Rad, Hercules, CA, USA). The cuvettes were immediately returned to the chamber, 500 µL of BHI broth was added to the competent cells, and the cells were incubated at 37 °C for ~24 h. The cells were then plated on BHI agar with 5 µg/mL erythromycin and incubated anaerobically at 37 °C for 5–7 days.

### 4.5. Bacterial Coaggregation Assay

The coaggregation between *F. alocis* (wild-type and ∆*srtA* mutant strains) and other oral bacteria with varying pathogenic potential (*F. nucleatum*, *T. forsythia*, *P. gingivalis*, *S. gordonii,* and *A. actinomycetemcomitans*) was performed as described earlier with minor changes [63]. Briefly, stationary-phase cultures of bacterial strains grown in their respective media were harvested by centrifugation. Bacterial cells were washed twice in coaggregation buffer (Tris-buffered saline, pH 7.5 containing 0.1 mM CaCl_2_) and normalized to an OD_600_ of 1.0 (~1  ×  10^9^ CFU/mL). Then, 1 mL aliquots of bacterial cell suspensions were vortex-mixed in glass tubes for a few seconds. The coaggregation percentage was determined after 30 min of incubation at room temperature without agitation. A total of 0.5 mL of the upper suspension was transferred to a cuvette, and the absorbance was measured at 600 nm. Coaggregated bacteria were photographed in a 24-well plate. The results represent the means of three independent experiments done in triplicate.

### 4.6. Biofilm Formation Assay

The in vitro biofilm assay was performed as described earlier, with minor modifications [21]. To grow monospecies biofilms, overnight bacterial cultures were diluted to OD_600_ ~0.05 in their respective media and 200 µL aliquots of diluted cultures were transferred to sterile, 96-well polystyrene plates (Greiner Bioscience, Monroe, NC, USA). Two different methods were used to grow dual-species biofilms: (1) 100 μL of each bacterial culture (OD_600_ ~0.05) was mixed, incubated for 10 min at 37 °C, and then transferred to a 96-well plate; (2) 100 μL of cultures of *F. nucleatum*, *P. gingivalis*, *S. gordonii*, *T. forsythia*, or *A. actinomycetemcomitans* (OD_600_ ~0.05) were placed on top of 100 μL of *F. alocis* cultures (OD_600_ ~0.05), which had been grown in 96-well plates for 24 h at 37 °C. All plates were incubated at 37 °C inside the anaerobic chamber. After 48 h, free-floating cells were aspirated, and the attached cultivated biofilms were gently washed with PBS (phosphate-buffered saline) and subsequently stained with 0.5% crystal violet for 30 min. The unbound dye was completely removed by washing with PBS several times. For quantitative analysis, biofilms were destained with ethanol/acetone mix (80:20) for 15 min and optical density at 580 nm was measured using xMark^TM^ microplate spectrophotometer (Bio-Rad Laboratories, Hercules, CA, USA). BHI broth without cells was used as blank control. The results were expressed as an average with standard deviations from four independent experiments performed in quadruplicate.

### 4.7. Collagen Binding Assay

For the binding assay [64], Greiner medium-binding 96-well plates (#655001) were coated overnight at 4 °C with collagen and fibronectin (50 μL of 1 mg/mL type I collagen, #354236, Corning Life Sciences, Corning, NY, USA; 50 μL of 200 μg/mL human plasma fibronectin, FC010, Millipore Sigma, Burlington, MA, USA). The next morning, the plates were washed with PBS. An overnight culture of *F. alocis* wild-type and ∆*srtA* mutant strains was centrifuged, washed, and resuspended in PBS to an OD_600_ of 1.0 (~10^9^ bacterial cells/mL). Then 100 μL of bacterial suspension was added to the coated plates and incubated at 37 °C. After 4 h incubation, nonadherent bacteria were removed by washing the cells with PBS. The collagen and fibronectin-bound bacteria were detached with 0.25% trypsin (Sigma–Aldrich), appropriately diluted, and plated on BHI agar, then incubated anaerobically for 5–7 days at 37 °C to enumerate the bound bacteria. Further, the percentage of bacterial binding was determined by comparing the CFU/mL of adherent bacteria for each strain with the input numbers. Each assay was performed in triplicate and repeated three times. All the steps of this assay were conducted inside the anaerobic chamber.

### 4.8. Air Sensitivity of Wild-Type F. alocis and *Δ*srtA Mutant

The air sensitivity of *F. alocis* strains was determined by exposing BHI agar-plated bacterial cultures to air [∼20% (*v*/*v*) oxygen] [19]. Briefly, overnight-grown (OD_600_ ∼0.24) liquid cultures of *F. alocis* wild-type and Δ*srtA* mutant strains were serially diluted, and appropriate dilutions were plated on BHI agar. The plates (except for input/time point zero) were moved from the anaerobic chamber to outside in the air at room temperature. At selected time points (1 h, 2 h, and 3 h), subsets of the plates were put back into the chamber, incubated at 37 °C and colonies were counted after 5–7 days of incubation. Percent survival was calculated as the ratio of CFU/mL of the air-exposed bacteria compared to the unexposed input. BHI broth and BHI agar plates used in this experiment had no cysteine. Experiments were done in triplicate and repeated at least three times independently.

### 4.9. Epithelial Cell Culture

Telomerase-immortalized gingival keratinocytes (TIGKs), a gingival epithelial cell line, were cultured in DermaLife K Serum-Free Keratinocyte Culture Medium (Lifeline Cell Technology, Frederick, MD, USA) at 37 °C in the presence of 5% CO_2_ [65]. The medium was supplemented with 0.5 ng/mL TGFα, 5 μg/mL insulin, 1 μM epinephrine, 5 μg/mL apo-transferrin, 100 ng/mL hydrocortisone, 0.4% bovine pituitary extract, and 6 mM L-Glutamine. The TIGK cell line is listed in the Cellosaurus database with the accession number CVCL_M095 and the ATCC catalog number is CRL-3397.

### 4.10. Cell Adhesion and Invasion Assays

The adhesion and invasion assays were performed as previously described, with minor modifications [19]. Briefly, overnight-grown cultures of *F. alocis* wild-type and ∆*srtA* mutant strains (OD_600_ ~0.25) were used to infect TIGK cells grown in 12-well plates (1.0 × 10^5^ cells per well) with a multiplicity of infection (MOI) of 100 (1.0 × 10^7^ CFU/mL) for 1 h at 37 °C. After 1 h incubation, loosely bound bacteria were removed by washing with PBS. The TIGK cells were then detached and lysed using 0.25% trypsin (Sigma–Aldrich) and 0.025% triton X-100 (Sigma–Aldrich), serially diluted and plated on BHI agar to count the attached and invaded bacteria. For the invasion assay, following 1 h of infection, extracellular bacteria were killed by exposure to 200 μg/mL metronidazole (Sigma–Aldrich) for 1 h. The TIGK cells were then washed, detached, lysed, serially diluted, and plated on BHI agar. Plates from both adhesion and invasion assays were incubated anaerobically at 37 °C for 5–7 days, after which the percentage of adherent and invaded bacteria was determined by comparing the CFU/mL of adherent and invaded bacteria with input numbers. The experiment was done in triplicate and repeated three times. The assay was performed entirely within the anaerobic chamber due to the sensitivity of the ∆*srtA* mutant to air.

### 4.11. Preparation of Protein Samples for Liquid Chromatography-Tandem Mass Spectrometry (LC–MS/MS) Analysis

*F. alocis* wild-type and ∆*srtA* mutant cells were separated into cell wall and extracellular medium fractions using a modified version of our previously described method [63]. Briefly, cells from overnight-grown bacteria (50 mL, OD_600_ of 0.25), were centrifuged to obtain the supernatant, representing the secreted culture medium and the cell pellet. The cell pellets were washed once with SMM buffer (0.5 M sucrose, 10 mM MgCl_2_, and 10 mM maleate, pH 6.8) and then treated with lysozyme (1 mg/mL in SMM buffer) for 3 h at 37 °C with constant rotation. After lysozyme treatment, soluble cell wall fractions were separated from the pelleted protoplasts by centrifugation. The proteins from both the culture medium and cell wall fractions were then precipitated with 100% TCA (*w*/*v*) and washed with acetone. The protein pellets were dissolved in 8 M urea/50 mM ammonium bicarbonate (pH 7.8), reduced with 5 mM DTT (37 °C for 1 h), then alkylated with 15 mM iodoacetamide for 30 min in the dark at room temperature. The reaction mixture was diluted with three volumes of 50 mM ammonium bicarbonate to lower the urea concentration. Proteins were digested overnight at 37 °C with mass spectrometry-grade trypsin (1:30, *w*/*w*) (trypsin gold, #V5280, Promega), under constant slow rotation. The resulting peptides were dried in a Speed Vac (Savant Instruments, Waltham, MA, USA) at room temperature, desalted using the C_18_ ZipTip pipette tips (Millipore Corporation, Burlington, MA, USA) and reconstituted in 20 µL of 1% formic acid before being transferred to sample vials for MS analysis. All chemicals, including lysozyme, TCA, acetone, urea, ammonium bicarbonate, DTT, iodoacetamide and formic acid, were purchased from Sigma–Aldrich.

### 4.12. LC–MS/MS Data Acquisition

LC–MS/MS data acquisition was performed using a data-dependent acquisition (DDA) method on an Orbitrap Exploris 240 mass spectrometer (Thermo Fisher Scientific), coupled with a Vanquish Neo UHPLC liquid chromatography system (Thermo Fisher Scientific) [66]. Peptide samples were separated using a DNV PepMap Neo column (1500 Bar,75 μm × 150 mm, 2 μm C18 from Thermo Fisher Scientific) at a flow rate of 300 nL/minute. All separations were carried out with the column oven set to 50 °C. The mobile phases A and B were 0.1% formic acid in water and in acetonitrile (Sigma Aldrich), respectively. A 135-min gradient was employed as follows: 0–90 min, 2–25% B; 90–120 min, 25–40% B; 120–135-min, 80% B. Each sample was separated using a trap-and-elute configuration.

The mass spectrometry instrument settings were as follows: full MS scans covered an *m*/*z* range of 350–1500. The resolutions for full-scan and ddMS2 were set at 6000 and 15,000 at *m*/*z* 200, respectively. For the DDA setting, the most intense ions captured within a 2 s cycle time were selected. The AGC target was set to 300% charges with a maximum injection time of 35 ms. Peptide fragmentation was performed in HCD at 28% collision energy. Dynamic exclusion was enabled (60 s). A source voltage of 2000 V and an ion transfer tube temperature of 250 °C were used for all experiments.

### 4.13. LC–MS/MS Data Analysis and Statistics

Raw data obtained from the LC–MS/MS analysis were processed using Proteome Discoverer software (v2.5.0.400, Thermo Fisher Scientific). The spectra were searched against the *F. alocis* ATCC 35896 proteome (1719 entries) using the UniProt database. Precursor and Protein Group false discovery rate (FDR) thresholds were set at 1%. Label-free quantification of proteins was performed using the summed abundances of all unique peptides, considering only precursors that passed the FDR thresholds. Trypsin was set as the digestion enzyme, allowing up to two missed cleavages and a minimum peptide length of six amino acids. Oxidation (+15.995 Da) of methionine and acetylation (+42.011 Da) of the N-terminus were set as variable modifications. Carbamidomethylation (+57.021 Da) of cysteine was set as a static modification. Relative quantification was carried out using the Feature Mapper and Precursor Ions Quantifier nodes.

### 4.14. Cloning, Expression, and Purification of Recombinant *SrtA* (*SrtA*_*∆N72*_)

The sortase gene *FA1364* (excluding the N-terminal 216 bases) was PCR amplified using *F. alocis* genomic DNA and a pair of gene-specific primers (Fa1364-pet102-for and Fa1364-pet102-rev, Table 3). The PCR product was then cloned into the expression vector pET102/D-TOPO by using a Champion pET102 Directional TOPO expression kit (Life Technologies, Waltham, MA, USA) as per the manufacturer’s instructions [67]. The DNA sequence of the sortase in the recombinant plasmid pET102-*FA1364* was confirmed by sequencing and then transformed into *E. coli* BL21 Star^TM^ (DE3) competent cells for expression. *E. coli* BL21 cells carrying the recombinant plasmid were grown to OD_600_ ~0.6 at 37 °C in LB medium containing 100 μg/mL of ampicillin. Expression was induced with 1 mM IPTG (Sigma Aldrich) at 37 °C for 5 h. Once expressed, the protein was purified under native conditions using Ni-NTA resin according to the manufacturer’s instructions (Qiagen, Germantown, MD, USA). The purified protein fractions were subsequently dialyzed twice against 1 L of dialysis buffer containing 150 mM NaCl, 50 mM Tris-HCl, pH 7.5 at 4 °C and stored at −80 °C in 10% glycerol in aliquots [67].

### 4.15. Site-Directed Mutagenesis of Recombinant Plasmids

Site-directed mutagenesis (H128A, C191A, and R200A) was performed using the QuikChange II XL site-directed mutagenesis kit (Agilent Technologies Inc., #200521, Santa Clara, CA, USA) following the manufacturer’s instructions. Briefly, the recombinant plasmid DNA (pET102-*FA1364*) served as a template for PCR amplification with *Pfu* DNA polymerase, using forward and reverse primers that flank six codons on either side of the target mutation site (Table 3). After PCR, the amplified plasmids were digested overnight at 37 °C with DpnI (New England Biolabs, Ipswich, MA, USA) to remove methylated DNA from the original DNA template. The digested plasmids were transformed into *E. coli* XL10-Gold ultracompetent cells. Mutant plasmids were verified by sequencing and subsequently transformed into *E. coli* BL21 for protein expression.

### 4.16. In Vitro Recombinant *SrtA* Activity Assay

The in vitro activity of the wild-type and mutant SrtA_△N72_ enzymes was tested using a standard FRET (Fluorescence resonance energy transfer)-based cleavage assay [54,56]. The activity assay employed a synthetic peptide substrate Dabcyl-RHLPKTGDG-Edans (GenScript Inc., Piscataway, NJ, USA), which includes the sorting motif LPKTG with Dabcyl (4-(4-dimethylaminophenylazo) benzoic acid) at the N-terminal and Edans (5-[(2-aminoethyl) amino] naphthalene-1-sulfonic acid) at C-terminal. In this assay, when a peptide labeled with a fluorophore (Edans group) and a quencher (Dabcyl group) is cleaved by a sortase enzyme, the fluorophore is released from the quencher, resulting in an increase in fluorescence (measured as relative fluorescence unit or RFU). The reactions were performed in a 96-well black, clear-bottom plate (Greiner Bioscience) with a final volume of 200 μL containing 150 mM NaCl, 5 mM CaCl_2_, 50 mM Tris–HCl, at pH 7.5, either 5 or 10 µM wild-type or mutant SrtA enzyme, and 20 μM of peptide substrate. The reactions were incubated for 1 h at 37 °C. Peptide cleavage was quantified by measuring the increase in RFUs using a Synergy H1 microplate reader (BioTek, Santa Clara, CA, USA) with excitation at 350 nm and emission at 495 nm. As a control, peptides were incubated without the enzyme, and the RFUs of the test samples were normalized accordingly. All experiments were performed in triplicate across three biological repeats.

### 4.17. Bioinformatics Analysis

The nucleotide sequence of the *FA1364* gene from *F. alocis* ATCC 35896 and sequences of various sortase proteins were obtained from the NCBI database. Sortases were aligned using Clustal Omega 2.0 [68]. The transmembrane domain of FA1364 was predicted using the TMHMM 2.0. The three-dimensional (3D) structure of sortase protein was predicted, modeled, and analyzed by using the online software AlphaFold 3.

### 4.18. Statistical Analysis

All assays were performed in triplicate for each condition and repeated at least three times unless otherwise stated. Error bars represent the standard deviations from the means. GraphPad Prism (version 11.0) was used for all statistical analyses. Significant differences were identified using the student’s unpaired *t*-test and one-way or two-way analysis of variance (ANOVA) followed by Fisher’s LSD post hoc test.

## 5. Conclusions

In this study, we have shown that *F. alocis* FA1364 protein functions as a housekeeping sortase A (SrtA) and its sorting signal includes a LPKTG motif. SrtA is essential for the appropriate cell-wall localization of FA1006, FA1336, FA1424, and FA1750 proteins. The *srtA*-deficient mutant demonstrated severely decreased binding to collagen and host cells, biofilm formation, bacterial coaggregation activity, and survival under air exposure. Given the apparent importance of SrtA in *F. alocis* virulence, FA1364 may serve as a potential therapeutic target for managing periodontal diseases. Furthermore, a comprehensive investigation of the individual SrtA-anchored surface proteins may uncover new adhesion factors and offer new opportunities to interfere with virulence in *F. alocis*.

## Figures and Tables

**Table 1:** The putative substrates of *F. alocis* SrtA.

Protein Name	Mass (kDa)	Annotation	Predicted Conserved Domains (CDs)	E-Value for CDs
FA1006	89.9	Cna B-type domain -containing protein	Pfam05738 (CnaB-type domain)	7.64 × 10^−33^
FA1336	83.8	Cna B-type domain -containing protein	Pfam05738 (CnaB-type domain)	7.43 × 10^−18^
FA1424	205.7	SdrD B-like domain -containing protein	Pfam17210 (SdrD B-like domain)	3.15 × 10^−6^
FA1750	98.2	Cna B-type domain -containing protein	Pfam05738 (CnaB-type domain)	4.44 × 10^−19^

**Table 2:** Bacterial strains and plasmids used in this study.

Bacterial Strain or Plasmid	Genotype and Description	Reference/Source
**Bacterial Strains**		
*F. alocis* ATCC 35896	Wild-type strain	[59]
FLL101	Δ*FA1364*::*ermF* an isogenic derivative of *F. alocis* ATCC 35896	This study
*S. gordonii* DL1	Wild-type strain	[60]
*T. forsythia* ATCC 43037	Wild-type strain	[61]
*A. actinomycetemcomitans* ATCC 33384	Wild-type strain	ATCC
*P. gingivalis* ATCC 33277	Wild-type strain	[59]
*F. nucleatum* ATCC 25586	Wild-type strain	[60]
*E. coli* Top 10	Used for cloning purpose	Invitrogen
*E. coli* BL21Star^TM^ (DE3)	Used as protein expression strain	Invitrogen
**Plasmids**		
pVA2198	Sp^r^, *ermF-ermAM*	[21]
pET102-TOPO	Ap^r^, C-terminal His-tag	Invitrogen
pET102-*FA1364*	Ap^r^, pET102 derivative expressing *FA1364*	This study

**Table 3:** Primers used in this study.

Primer	Sequence (5′-3′) ^a^
**Mutant construction**	
P1-Fa1364-up-for	TTATCGGAAGGAATCAGCGG
P2-Fa1364-erm-rev	CGGGCAATTTCTTTTTTGTCATGAACTATCTTCCTTTTATCTC
P3-erm-for	ATGACAAAAAAGAAATTGCCCGTTCGTTTTACGGGTCAGCACTT
P4-erm-rev	GATTATTCCCTCCAGGTACTACGAAGGATGAAATTTTTCA
P5-Fa1364-erm-for	TCGTAGTACCTGGAGGGAATAATCTAGACAGATTTTCTTTGACGG
P6-Fa1364-dn-rev	CCAGTTCATGGTTTCTCTTAAA
Protein expression	
Fa1364-pet102-for	CACCAAAGAGTATGAAAACGCAATC
Fa1364-pet102-rev	TTGTAATTCTCTTACTTCCGC
**Site-directed mutagenesis**	
Fa1364-H128A-for	GTAAGTGTAATCGCAGGAgctCGTGGCGGATACAATGGA
Fa1364-H128A-rev	TCCATTGTATCCGCCACGagcTCCTGCGATTACACTTAC
Fa1364-C191A-for	AAATTGATTTTGTTATCTgctCATCCTTATCCAAAAAAT
Fa1364-C191A-rev	ATTTTTTGGATAAGGATGagcAGATAACAAAATCAATTT
Fa1364-R200A-for	TATCCAAAAAATACCGACgctATTTTAATTTATTCCAAA
Fa1364-R200A-rev	TTTGGAATAAATTAAAATagcGTCGGTATTTTTTGGATA

^a^ Complementary bases are underlined.

## Data Availability

The original contributions presented in this study are included in the article. Further inquiries can be directed to the corresponding author.

## Supplementary Materials

The following supporting information can be downloaded at: https://www.mdpi.com/article/10.3390/ijms27114783/s1.
