# Supplementary material for: Functional Characterization of a Putative Sortase FA1364 in Filifactor alocis
Source: Int J Mol Sci. 2026 May 26;27(11):4783. doi: 10.3390/ijms27114783 (PMC13257024; doi:10.3390/ijms27114783)
Supplement: Supplementary file 1 [file ijms-27-04783-s001.zip › ijms-4239693-supplementary.pdf]

A

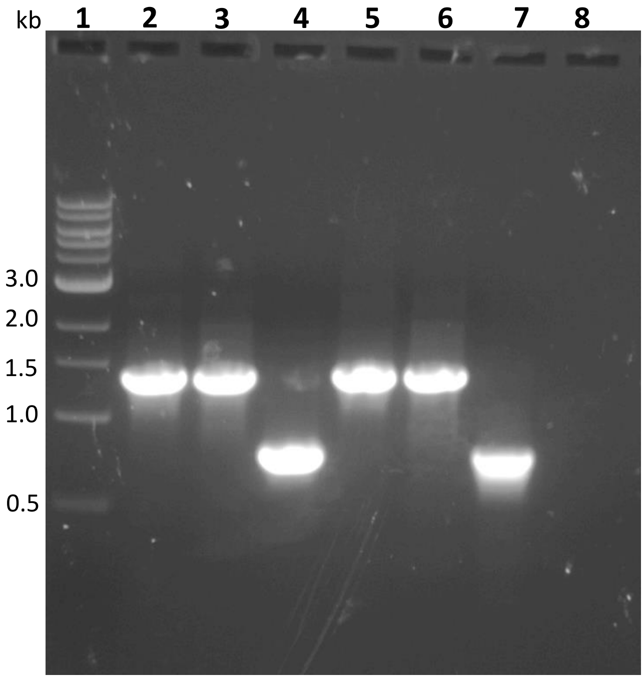

Lane 1-Molecular weight marker (1 kb DNA ladder)
Lane 2-PCR amplicon from Δ*srtA* mutant colony 1 (primers P1 and P4, 1.4 kb)
Lane 3-PCR amplicon from Δ*srtA* mutant colony 1 (primers P3 and P6, 1.4 kb)
Lane 4-PCR amplicon from Δ*srtA* mutant colony 1 (primers P3 and P4, 819 bp)
Lane 5-PCR amplicon from Δ*srtA* mutant colony 2 (primers P1 and P4, 1.4 kb)
Lane 6-PCR amplicon from Δ*srtA* mutant colony 2 (primers P3 and P6, 1.4 kb)
Lane 7-PCR amplicon from Δ*srtA* mutant colony 2 (primers P3 and P4, 819 bp)
Lane 8- No *ermF* PCR amplicon from wild-type *F. alocis* (primers P3 and P4)

B

TTATCGGAAGGAATCAGCGGAACTTATGCAAGTGCGATAGAGTTTGCAAAACTATTCGAAGAAGATAATTTCAAGATACAGATATTGCC
GACCAAAGGTGTTTCTATGATTATAGGGCATCCGGCATTGGAGATGGCAAAGACAGCAAGAGAAACAGGTGATTTTGTGCAAACAGTTT
CACTTGGCAAACAACTTTTGCAGAGAACATGTGTATATTTTGCAGTAGAGAGTTTGAAATACCTTATTTTGGGAGGAAGAATTTCAAAA
GTTGAGGGAACAATCGCAGAAATGCTTGATATTAAACCGATTGTCGGAGTTAACCCGGAAGACGGGACATTGTCCTCTGTGGGCAAAGT
GAGAGGACGAAAGCGTTCTATCAAAAAAGTTTTGGAAACATTCAAAGATGAAATCGGTACACAGGAAGTAGACAAAATTTTTGTCGTAC
ATGGCGAACGGATGGAAGATGCAGAATATTTACAGGAACTGTTTCAGCAGGAATATCCGAATGCAGGAACGGAAATTCACTCCCTGAGT
GCTTTGCTGACTGTACACACAGGACCCGGAACAATCGGTGCAGCAATATTTTTGAAATAAGAGATAAAAGGAAGATAGTTCATGACAAA
AAAGAAATTGCCCGTTCGTTTTACGGGTCAGCACTTTACTATTGATAAAGTGCTAATAAAAGATGCAATAAGACAAGCAAATATAAGTA
ATCAGGATACGGTTTTAGATATTGGGGCAGGCAAGGGGTTTCTTACTGTTCATTTATTAAAAATCGCCAACAATGTTGTTGCTATTGAA
AACGACACAGCTTTGGTTGAACATTTACGAAAATTATTTTCTGATGCCCGAAATGTTCAAGTTGTCGGTTGTGATTTTAGGAATTTTGC
AGTTCCGAAATTTCCTTTCAAAGTGGTGTCAAATATTCCTTATGGCATTACTTCCGATATTTTCAAAATCCTGATGTTTGAGAGTCTTG
GAAATTTTCTGGGAGGTTCCATTGTCCTTCAATTAGAACCTACACAAAAGTTATTTTCGAGGAAGCTTTACAATCCATATACCGTTTTC
TATCATACTTTTTTTGATTTGAAACTTGTCTATGAGGTAGGTCCTGAAAGTTTCTTGCCACCGCCAACTGTCAAATCAGCCCTGTTAAA
CATTAAAAGAAAACACTTATTTTTTGATTTTAAGTTTAAAGCCAAATACTTAGCATTTATTTCCTGTCTGTTAGAGAAACCTGATTTAT
CTGTAAAAACAGCTTTAAAGTCGATTTTCAGGAAAAGTCAGGTCAGGTCAATTTCGGAAAAATTCGGTTTAAACCTTAATGCTCAAATT
GTTTGTTTGTCTCCAAGTCAATGGTTAAACTGTTTTTTGGAAATGCTGGAAGTTGTCCCTGAAAAATTTCATCCTTCGTAGTACCTGGA
GGGAATAATCTAGACAGATTTTCTTTGACGGTTTTATTCTGTGTGAAAGAAAATGCAATATCAAAAAAACTCTTATTTGTCCGCTTTAT
AAGCAAAATCGGTTCCAATAAGAGTTTTTTATTGCTCAAAAATCAAGAAGAACTGATATGAAAAAATAGAAGAAAAAAATTTTATTGGAT
AGGGGAACTAACAGTTTGAAATAACAGTTAGGAGAAAGTTAGAGCCGAAGATAGTGCATGGAAAACTCGGGAATAGGGACTTTAAAGAA
GGAAAAAGATATCCTATATTCTGAAATTGCAGACATTTGAAAAGAAGTTGAACAATTGGATACATTCAGAAGCTGTATAGAGAAATGAC
TGCAAGGAAACAGAGAACTAACACAGGTCAACAAGTAACAGTTAGAGATCTATTAGAGCTATAAATAGAAGTTGAATTAGAGTATAATA
ATGATAATTGTTGAATGAAATCAATTTGAAGTTGTGGAGATAACAAAAATGAATATTATACTAAAAAATACCCCTGAAACATACAAAAA
AATGGTGGATGAAATCATACCGCCATTACTTGAACTGTTGCAGGAATTGACTTTACTGGAAGAAGAAATTTTTAAGAGAAACCATGAAC
TGGATGTTGAAAAATCTATCTTGAATATTCCT

Supplementary Figure S1. Confirmation of the *srtA* gene replacement with *ermF* in the *F. alocis* Δ*srtA* mutant by (A) PCR and (B) sequencing. (A) Different sets of primers were used for PCR amplification using genomic DNA template from the Δ*srtA* mutant or the wild-type strains. Size of the upstream fragment, *ermF* cassette and downstream fragment is 615 bp, 819 bp and 616 bp respectively. Both colonies of the mutant showed amplification of the *ermF* fragment (lanes 4 and 7). Wild-type strain showed no amplification as a control (lane 8). (B) *ermF* and *F. alocis* sequences are shown in red and black respectively.

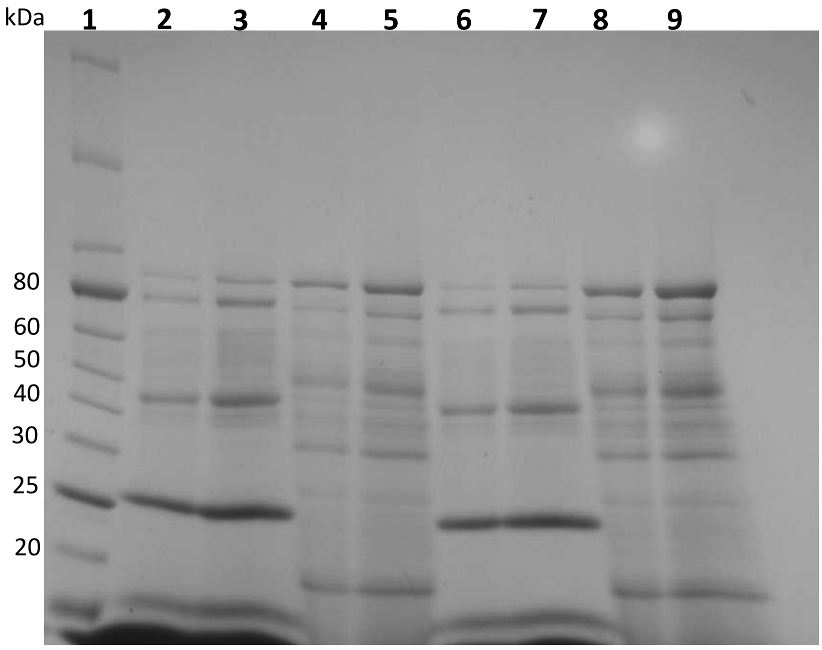

Lane 1-Molecular weight marker
Lane 2-Wild-type *F. alocis* (W, 2.5 µg)
Lane 3-Wild-type *F. alocis* (W, 5 µg)
Lane 4 Wild-type *F. alocis* (M, 2.5 µg)
Lane 5-Wild-type *F. alocis* (M, 5 µg)
Lane 6-Δ*srtA* mutant (W, 2.5 µg)
Lane 7-Δ*srtA* mutant (W, 5 µg)
Lane 8-Δ*srtA* mutant (M, 2.5 µg)
Lane 9-Δ*srtA* mutant (M, 5 µg)

Supplementary Figure S2. SDS-polyacrylamide gel electrophoresis (PAGE) analysis of the cell wall (W) and extracellular medium (M) fraction proteins from *F. alocis* wild-type and Δ*srtA* mutant strains. Purified proteins were analyzed on the 3%–12% tris-glycine gel in reducing sample buffer. Molecular weight markers (in kilodaltons, kDa) are indicated on the left.
